# Inclusion of persons with disability in sport: part 1 – rights and challenges in Qatar

**DOI:** 10.1136/bjsports-2022-106224

**Published:** 2022-09-16

**Authors:** Sanaa Taha Al-Harahsheh, Kamilla Swart, Josélia Neves, Sabika Shaban

**Affiliations:** 1 World Innovation Summit for Health, Research and Content Department, Qatar Foundation, Doha, Qatar; 2 College of Science and Engineering, Hamad Bin Khalifa University, Doha, Qatar; 3 College of Humanities and Social Sciences, Hamad Bin Khalifa University, Doha, Qatar; 4 College of Islamic Studies, Hamad Bin Khalifa University, Doha, Ad Dawhah, Qatar

**Keywords:** Sport, Education, Physical activity, Public health, Recreation

Sport is considered a powerful tool to foster social inclusion and improve the well-being of persons with disabilities (PWDs). While it can place people on equitable social footing,^1^ PWDs remain under-represented in sport and physical activities compared with their peers without disabilities.

The participation of PWDs in sport is influenced by the type and severity of disability. Those with learning disabilities or with profound and multiple disabilities have the lowest participation levels.^1^ Globally, disability rates are rising dramatically, presently estimated at over 1 billion people—including 190 million people (3.8%) who experience significant difficulties in functioning.^2^ In Qatar, census data estimates 1.2% of the population have a disability, with 232 athletes registered at the Qatar Paralympic Committee (QPC).^3^ However, it is important to note that defining and operationalising disability remains a challenge despite significant progress in measurement. Qatar has around 1.3 million employees, mostly young, healthy men, and uses a narrow definition of disability when estimating the number of PWDs. Therefore, the Washington Group Questions should be adopted in the future to measure PWDs more accurately.^4^

Over the past few decades, the State of Qatar has achieved tangible progress in catering to the needs of PWDs. For example, various projects, initiative and programmes that accommodate to the needs of PWDs, while protecting their basic human rights were developed. Furthermore, the QPC is not only committed to enabling para-athletes to achieve sporting excellence but also to developing sport opportunities for all PWDs in Qatar (beginner to elite).^5^ This editorial reflects on how sport and physical activities affect PWDs in Qatar and the obstacles to their participation.

## Disability rights: inclusion and sport

As a form of social engagement, sport participation is a fundamental human right supported by many international and national policies, including the Universal Declaration of Human Rights (1948), the UN Convention on the Rights of Persons with Disabilities (2006), the UNESCO’s KAZAN Action Plan, the 2030 Agenda for Sustainable Development, the Qatar Law No. 2 for 2004 and the 2019 Doha Declaration (See table 1). Qatar, like every state, has an obligation to take proactive and appropriate measures to ensure that PWDs participate in all aspects of society on an equitable basis. The rights of PWDs were also emphasised in the Qatar National Vision 2030, and in the first (2011–2016) and second (2018–2022) Qatar National Development Strategies. Although Qatar has taken important steps to promote and protect the rights of PWDs, challenges persist and PWDs remain under-represented in all forms of cultural life, including sport.

**Table 1:** A summary of the international and national policies supporting the rights and needs of persons with disabilities (PWDs)

International and national policies for PWDs	Overview
The Universal Declaration of Human Rights (1948)	The right to disability rights is established in the Universal Declaration of Human Rights (1948). Play, sport and physical activity are implicitly recognised as rights in the Universal Declaration.
The UN Convention on the Rights of Persons with Disabilities (2006)	As stipulated in Article 30 of the UN’s (2006) CRPWDs, signatories ‘…recognize the right of persons with disabilities to participate on an equal basis with others in cultural life’ (defined as recreation, leisure, the arts, sport and tourism). The article highlights the importance of treating PWDs equally, and states should improve access to and support the inclusion of PWDs in recreational, leisure and sporting activities (article 4).
UNESCO’s KAZAN Action Plan	The Action Plan states that to reduce inequalities at the national and international levels, inclusive sport policies are necessary. Therefore, physical education, physical activity and sport should be at the core of all national and international sport policies.
The 2030 Agenda for Sustainable Development	The 2030 Agenda for Sustainable Development recognises sport as an important enabler for sustainable development and peace, and a vital tool for youth, women and communities to reach health, education and social inclusion objectives.
Qatar Law No. 2 for 2004	Qatar Law No. 2 of 2004 establishes a comprehensive legal framework for PWDs, including 14 articles that ensure their care and legal protection, so they can exercise their rights equally with everyone else. In 2008, Qatar had ratified the Convention on the Rights of Persons with Disabilities and in April 2015 it had adopted a law on persons with disabilities, which covered the rights contained in the Convention, and had established the National Committee with representatives of persons with disabilities whose mandate was to monitor compliance with the Convention.
The 2019 Doha Declaration	The movement towards inclusion in Qatar has gained new impetus with the formal commitment to enact the 2019 Doha Declaration, ‘a core reference point internationally for policy development about human rights and sustainable development in the context of disability’. This Declaration was the landmark outcome of the Doha International Conference on Disability and Development, held on 7–8 December 2019, under the guidance and patronage of Her Highness Sheikha Moza bint Nasser, Founder of Qatar Foundation for Social Work and its affiliated civil society centres. This Declaration, that sets forth 11 recommendations to make Qatar (and other countries who may come to commit to it) actively adjusting itself to the needs of PWDs, is an incentive to all those who are already on the ground working towards making Qatar a more welcoming and inclusive country.

## Disability and sport

In this editorial, the term disability sport is used to refer to all sport, physical activity, recreation and leisure for and involving PWDs, including adaptive sport or parasport.^6^ Adapted physical activity is found in different application areas, including inclusive and specialised physical education, competitive sport and recreational physical activity; and can be placed on par with mainstream modalities.

## Adapted modalities

The QPC is responsible for managing participation at the Paralympics and other international competitions as well as for providing opportunities for PWDs in Qatar to participate in sport at all levels. The Paralympics is an international sporting event in which para-athletes compete in six disability groups (amputee, cerebral palsy, visual impairment, spinal cord injuries, intellectual disability and ‘les autres’—any disability that does not fall into any of the other categories). The Paralympic movement recognises 10 impairment types and para-athletes are divided further into classes depending on the type and extent of their disabilities.^7^

The International Paralympic Committee serves as an umbrella organisation that represents all sports with disability. It supports 200-plus members, including 182 national Paralympic committees (of which QPC is 1), 4 Paralympic sport federations (boccia, sitting volleyball, wheelchair basketball and wheelchair rugby) and 4 international organisations of sport for disability that focus on grassroots sport development, viz CP-ISRA (cerebral palsy), IBSA (vision impairment), IWAS (wheelchair and amputee) and Virtus (intellectual impairment), among others (see online supplemental file).

10.1136/bjsports-2022-106224.supp1 Supplementary data

## Benefits of sport

The benefits of sport are universal for all including those with disabilities. Through sport, PWDs can advance social inclusion, health and life skills.^8^ It fosters social and psychological well-being by providing opportunities for friendship, a sense of self and meaning and purpose in life. It positively affects the way PWDs perceive their bodies, leading to better mood states, less stress and increased self-esteem.^9^ It develops social belonging, improved communication and better coping with the stigma associated with disability.^10^ Despite these universal benefits, PWDs still face various barriers to participating in sport and other physical activities.

## Challenges and constraints

Factors that hinder sport participation by PWDs are summarised here into three categories.^9^ Intrapersonal constraints involve psychological conditions that are internal to the individual (eg, personality, attitudes, mood, stress and perceived self-skill). Interpersonal constraints arise from interactions with other members of society. Structural constraints include factors such as the lack of opportunities and accessibility or the cost of activities that result from external conditions in the environment. Additionally, the global COVID-19 pandemic has had a significant influence on sport and physical activity, leading to the closure of sport and physiotherapy facilities and spaces. It also resulted in the unprecedented delay of the Olympic and Paralympic Games, and the cancellation of athletic activities at every level, directly limiting the social opportunities and advantages of global, regional and local sporting events for PWDs.^11^

In Qatar, it is difficult to produce evidence-based policies due to the lack of adequate data on disabilities. Pockets of data gathered by scattered entities, combined with dated or scant figures from previous censuses, have led to the disability community failing to receive due support, whether at the local or global scale.^12^

Recognising these barriers and challenges creates an undeniable opportunity to effect change. Qatar has progressed through several formative stages and is currently ripe with prospects for advancing to an inclusive nation. Moving forward, evidence should be generated to better describe the current state of disability and inclusion in sport within Qatar. National and international organisations must also work together to increase the opportunities and access of PWDs to sporting activities. Governments have an important role to play in supporting such initiatives, increasing funding and promoting awareness for the importance of sport participation by PWDs.
