# Supplementary material for: Inclusion of persons with disability in sport: part 1 – rights and challenges in Qatar
Source: Br J Sports Med. 2022 Sep 16;56(22):1257–8. doi: 10.1136/bjsports-2022-106224 (PMC9626912; doi:10.1136/bjsports-2022-106224)
Supplement: Supplementary data [file bjsports-2022-106224supp001.pdf]

Supplementary File

Further information on the International Paralympic Committee (IPC) and an overview of all other international organizations providing sport for persons with disabilities (PWDs)

Organization	Overview
IPC	• Paralympic Games was initially known as the Stoke Mandeville Games<br>• It was organized in 1948 by a neurosurgeon, Sir Ludwig Guttmann, who had established the Stoke Mandeville Spinal Injuries Unit for rehabilitation purposes and became known as the father of the Paralympic movement.[1]<br>• It was only in 1989 that the IPC was founded as a collective voice for advocacy for the Paralympic movement based on previous cooperation of several international organizations intent on stabilizing and extending elite sport to PWD .[1,2]<br>• In 2008 the relationship between the International Olympic Committee and the IPC strengthened with the adoption of the "one city, one bid: model for the hosting of both the Olympic and Paralympic Games by the same organizing committee. [1,2]<br>• Ten impairment types recognized by the Paralympic movement include impaired muscle power, impaired passive range of movement, limb deficiency, leg length difference, short stature, hypertonia, ataxia, athetosis, vision impairment, and intellectual impairment.[3]<br>• IPC supports 15 International Sport Federations (11 of which are Olympic and Paralympic Federations: archery, badminton, canoe, cycling, equestrian, rowing, table tennis, taekwondo, triathlon, wheelchair curling and wheelchair tennis; while four are Paralympic Sport Federations: boccia, sitting volleyball, wheelchair basketball and wheelchair rugby<br>• IPC organizes the Paralympic Summer (22 sports) and Winter Games (6 sports) every four years after the Olympic Games. Two Paralympic sports that do not have Olympic counterparts are boccia (played by those who have significant physical impairments) and goalball **played exclusively by athletes who are blind or vision impaired.**<br>• Paralympic Games have grown immensely popular over the years; with the most recent Tokyo Games 2020+1 attracting the highest number of para-athletes (4403) from 161 countries [2]; despite the Covid-19 pandemic. Tokyo 2020+1 also had the largest number of women para-athletes (1853) participating ever.<br>• Based on a recent governance review of the IPC, it was agreed at that there would be a managed exit process of 10 IPC sports (alpine skiing, athletics, biathlon, cross country skiing, dance sport, ice hockey, power lifting, shooting, snowboarding and swimming) whereby the IPC would stop acting as the international federation for these sports by the end of 2026.[4] They will be governed by their respective international sport federations who have underscored that para-sport integration into their respective organizations demonstrates commitment to inclusivity and to the development of their sports.[4]
International Committee of Sports for the Deaf (ICSD)	• Initially known as the International Committee for Deaf Sports (ICSS) is the oldest international federation for PWDs. [1]<br>• Inaugural International Games for the Deaf was held in Paris in 1924<br>• operates on a four-year cycle, with the Winter World Games initiated a little later (1949) in comparison to the Summer Deaf Games<br>• IOC recognized the ICSS in 1951 and it became a founding member of the IPC . [1]<br>• In 1995 the ICSS withdrew from the IPC<br>• In 1996, the name of the Games was changed to "World Games for the Deaf" and then changed again in 2001 to "Deaf Olympics or "Deaflympics". [5]

Al-Harahsheh ST, *et al. Br J Sports Med* 2022; 56:1257–1258. doi: 10.1136/bjsports-2022-106224

	• Athletes who have a hearing loss of at least 55db in one ear are eligible to participate, and the use of any hearing instrument is not allowed.<br>• Flags and lights are used instead of whistles and pistols<br>• All the sports are played the same way as in the Olympics (unlike the Paralympics, which has different rules and regulations).[6]<br>• 24th edition of the Deaflympics Summer Games took place in May 2022 in Brazil, with 2412 (1647 and 765 women) participants from 73 countries[1] ,
International Stoke Mandeville Wheelchair Sports Federation (ISMWSF)	• Initially called the International Stoke Mandeville Games Federation (ISMGF)<br>• Formed as a result of the annual festivals of sport taking place which became the inception of the Paralympic Games phenomenon we know today [1]<br>• Participation was restricted to people with spinal cord lesions in its earlier years<br>• In 1990 ISMGF's name changed to ISMWSF
International Sports Organization for the Disabled (ISOD)	• Established in 1964 to serve amputees and 'les Autres'[1]<br>• Set out to provide for those PWD that were not represented in the other sport federations<br>• With more disability-specific sport organizations emerging in the 1970s such as the International Blind Sport Association (IBSA) and the Cerebral Palsy – International Sports and Recreation Association (CP-ISRA), ISOD started to lose its importance as a coordinating body for athletes outside the ISMGF framework<br>• In 2004 IMSWSF and ISOD merged to form the International Wheelchair and Amputee Sports Federation (IWAS). [1]
CP-ISRA	• Formed in 1978 to promote sport and recreational activities for people with cerebral palsy and related conditions such as those affected by strokes and traumatic brain injury [1]<br>• Takes responsibility for all athletes with cerebral palsy participating in the Paralympic Games<br>• Active in ascertaining the most appropriate elite sport opportunities for people with severe disabilities together with the IPC. [1]
International Association for Sport for Persons with Intellectual Disability (INAS-FID)	• International Association for Sport for Persons with Mental Handicap (INAS-FMH) was stablished in 1986 to provide sport for people with intellectual disability. [1]<br>• Its name changed in 1999 to INAS-FID<br>• Due to the challenges in classifying eligibility have caused problems between them and the IPC. [1]
International Special Olympics	• Founded in 1968 by Eunice Kennedy Shriver .[1]<br>• She started this movement with a backyard summer camp for children with intellectual disabilities at her home in 1962<br>• Led to the first international Special Olympics Games being held in Chicago in July 1968. [7]<br>• In 1998, the Special Olympics Unified Sports initiative was launched which brings together people with and without intellectual disabilities on the same teams.[7]<br>• Special Olympics World Games include summer and winter editions which alternate every two years. [7]<br>• Special Olympics Summer Games generally comprise 30 sports whereas the Winter Games include 6 sports. [7]<br>• 16th World Games will take place in Berlin in 2023 and is anticipated to attract 7,000 athletes from 190 nations.[7]<br>• IOC endorsed the Special Olympics in February 1998; and while the IOC tried to bring Special Olympics and INAS-FID together, the Special Olympics remains somewhat distant from the Paralympic Movement. [1]
IBSA	• Established in 1981 to serve blind and visually impaired athletes. [8]

Al-Harahsheh ST, *et al. Br J Sports Med* 2022; 56:1257–1258. doi: 10.1136/bjsports-2022-106224

	• Governing body for three Paralympic sports; viz. goalball, judo and blind football; as well as showdown, powerlifting, nine and tenpin bowling, chess and torball. [8]<br>• Also hosts its own World Games every four years, with the next edition scheduled for Birmingham in 2023 which will feature 1000 competitors from more than 70 nations participate in three Paralympic and eight non-Paralympic sports. [8]

Sources:

1. Bailey S. Athlete first: A history of the Paralympic movement [Internet]. Newy York: Wiley-Interscience; 2008 [cited 2022 Jul 24]. Available from: https://www.wiley.com/en-us/Athlete+First%3A+A+History+of+the+Paralympic+Movement-p-9780470724316

2. Brittain I. Key points in the history and development of the Paralympic Games. In: Brittain I, Beacom A, editors. The Palgrave Handbook of Paralympic Studies. London, UK: MacMillan; 2018. p. 125–49.

3. IPC classification - Paralympic categories & how to qualify [Internet]. International Paralympic Committee. [cited 2022 Jul 24]. Available from: https://www.paralympic.org/classification

4. Hernan. Tokyo 2020 sets the record for most athletes and women at a Paralympic Games [Internet]. International Paralympic Committee. 2021 [cited 2022 Jul 16]. Available from: https://www.paralympic.org/news/tokyo-2020-sets-record-most-athletes-and-women-paralympic-games

5. Williams M. FIS to take control of Para skiing disciplines, IBU to manage Para biathlon [Internet]. Sport Business. 2022 [cited 2022 Jul 16]. Available from: https://www.sportbusiness.com/news/fis-to-take-control-of-para-skiing-disciplines-ibu-to-manage-para-biathlon/?utm_source=newsletter&utm_medium=email&utm_campaign=London+PM_2022-07-14

6. McPhillips E. All about the Deaflympics. Audicus. 2018. [cited 2022 Jul 16]. Available from https://www.audicus.com/all-about-the-deaflympics/

7. Special Olympics. (n.d.a). History. [cited 2022 Jul 16]. Available from: https://www.specialolympics.org/about/history

8. IBSA. (n.d.a). History. [cited 2022 Jul 16]. Available from: https://ibsasport.org/about/who-we-are/history/
